# Correlation of Perfusion MRI and ^18^F-FDG PET Imaging Biomarkers for Monitoring Regorafenib Therapy in Experimental Colon Carcinomas with Immunohistochemical Validation

**DOI:** 10.1371/journal.pone.0115543

**Published:** 2015-02-10

**Authors:** Ralf S. Eschbach, Wolfgang P. Fendler, Philipp M. Kazmierczak, Marcus Hacker, Axel Rominger, Janette Carlsen, Heidrun Hirner-Eppeneder, Jessica Schuster, Matthias Moser, Lukas Havla, Moritz J. Schneider, Michael Ingrisch, Lukas Spaeth, Maximilian F. Reiser, Konstantin Nikolaou, Clemens C. Cyran

**Affiliations:** 1 Department of Clinical Radiology, Laboratory for Experimental Radiology, University Hospitals Munich, Ludwig-Maximilians-University Munich, Marchioninistrasse 15, 81377 Munich, Germany; 2 Department of Nuclear Medicine, University Hospitals Munich, Ludwig-Maximilians-University Munich, Marchioninistrasse 15, 81377 Munich, Germany; 3 Department of Nuclear Medicine, University Hospital Vienna, Medical University Vienna, Währinger Gürtel 18–20, 1090 Vienna, Austria; 4 Department of Clinical Radiology, Josef Lissner Laboratory for Biomedical Imaging, University Hospitals Munich, Ludwig-Maximilians-University Munich, Marchioninistrasse 15, 81377 Munich, Germany; 5 Department of Diagnostic and Interventional Radiology, University Hospital Tuebingen, Hoppe-Seyler-Strasse 3, 72076 Tuebingen, Germany; Universidade de São Paulo, BRAZIL

## Abstract

**Objectives:**

To investigate a multimodal, multiparametric perfusion MRI / ^18^F-fluoro-deoxyglucose-(^18^F-FDG)-PET imaging protocol for monitoring regorafenib therapy effects on experimental colorectal adenocarcinomas in rats with immunohistochemical validation.

**Materials and Methods:**

Human colorectal adenocarcinoma xenografts (HT-29) were implanted subcutaneously in n = 17 (n = 10 therapy group; n = 7 control group) female athymic nude rats (Hsd:RH-Foxn1^rnu^). Animals were imaged at baseline and after a one-week daily treatment protocol with regorafenib (10 mg/kg bodyweight) using a multimodal, multiparametric perfusion MRI/^18^F-FDG-PET imaging protocol. In perfusion MRI, quantitative parameters of plasma flow (PF, mL/100 mL/min), plasma volume (PV, %) and endothelial permeability-surface area product (PS, mL/100 mL/min) were calculated. In ^18^F-FDG-PET, tumor-to-background-ratio (TTB) was calculated. Perfusion MRI parameters were correlated with TTB and immunohistochemical assessments of tumor microvascular density (CD-31) and cell proliferation (Ki-67).

**Results:**

Regorafenib significantly (p<0.01) suppressed PF (81.1±7.5 to 50.6±16.0 mL/100mL/min), PV (12.1±3.6 to 7.5±1.6%) and PS (13.6±3.2 to 7.9±2.3 mL/100mL/min) as well as TTB (3.4±0.6 to 1.9±1.1) between baseline and day 7. Immunohistochemistry revealed significantly (p<0.03) lower tumor microvascular density (CD-31, 7.0±2.4 vs. 16.1±5.9) and tumor cell proliferation (Ki-67, 434.0 ± 62.9 vs. 663.0 ± 98.3) in the therapy group. Perfusion MRI parameters ΔPF, ΔPV and ΔPS showed strong and significant (r = 0.67-0.78; p<0.01) correlations to the PET parameter ΔTTB and significant correlations (r = 0.57-0.67; p<0.03) to immunohistochemical Ki-67 as well as to CD-31-stainings (r = 0.49-0.55; p<0.05).

**Conclusions:**

A multimodal, multiparametric perfusion MRI/PET imaging protocol allowed for non-invasive monitoring of regorafenib therapy effects on experimental colorectal adenocarcinomas *in vivo* with significant correlations between perfusion MRI parameters and ^18^F-FDG-PET validated by immunohistochemistry.

## Introduction

In recent years a broad range of novel molecular cancer therapeutics were introduced into clinical use [1–3]. Among these are anti-angiogenic therapies, like tyrosine kinase inhibitors, which have shown their effectiveness in the treatment of several malignancies including colorectal cancer [4–6]. Regorafenib, an oral multi tyrosine kinase inhibitor, showed *in vivo* anti-angiogenic and anti-proliferative effects in different experimental tumor models, such as breast cancer, renal cell carcinoma and glioblastoma and has proven effectiveness in the clinical treatment of metastatic colorectal cancer [7,8]. These new therapy regimes demonstrated significant effects on tumor angiogenesis and tumor metabolism, but often only subtle effects on tumor morphology, particularly in early stages of tumor treatment [9]. However, it has been shown, that established methods of monitoring cytotoxic tumor therapies, such as morphology-based Response Evaluation Criteria in Solid Tumors (RECIST) are not sufficiently sensitive for monitoring the early therapeutic effects of molecular anti-cancer agents to allow for a timely differentiation of responders from non-responders [10,11].

Functional imaging techniques including perfusion magnetic resonance imaging (MRI) and positron emission tomography (PET) can be applied for the assessment of physiological processes *in vivo* such as tissue microcirculation or glucose metabolism [12–15]. In multiparametric MRI protocols these functional surrogate markers of tumor metabolism are complementing state-of-the-art high resolution imaging of tumor morphology adding valuable information for a more comprehensive, non-invasive characterization of the tumor microenvironment [16,17]. Multiple experimental and clinical studies demonstrated the potential of contrast-enhanced perfusion MRI for the non-invasive assessment of anti-angiogenic treatment effects on several cancer entities as well as their potential applicability as non-invasive imaging biomarkers of therapy response [18–21]. However, controversy still exists not only on standardized protocols of data acquisition and analysis, but also with respect to the pathophysiologic correlate of altered tumor microcirculation under molecular cancer therapy [22–25]. A multiparametric characterization of treatment effects with the assessment of different aspects of tumor metabolism under therapy, e.g. glucose metabolism quantified by ^18^F-FDG PET, is of particular interest not only to acquire a multi-facetted chart of treatment effects but also to reveal potential interdependencies between the different acquired parameters of tumor pathophysiology. Multimodality hybrid imaging protocols such as MRI/PET are able to assess a number of functional imaging parameters in a one-stop-shop approach, including tumor perfusion and glucose metabolism, allowing for an intraindividual comparison and validation of the parameters.

Therefore, the hypothesis of our study was that a multimodality, multiparametric imaging protocol including perfusion MRI and ^18^F-FDG PET can be applied for monitoring the anti-angiogenic and anti-proliferative effects of regorafenib on experimental colon carcinomas *in vivo*. The purpose of our project was first, to investigate whether the acquired parameters of tumor microcirculation and glucose metabolism can be applied as non-invasive imaging biomarkers of therapy response, validated by immunohistochemistry, and second to evaluate a potential biological relationship between tumor microcirculation and tumor glucose metabolism by correlating the acquired parameters.

## Materials and Methods

### Animal model and experimental protocol

This study was carried out in strict accordance with the recommendations in the Guide for the Care and Use of Laboratory Animals of the National Institutes of Health. The protocol was approved by the Government of Upper Bavaria Committee for Animal Research (Gz.55.2–1–54–2532–33–10). Human colon cancer cells HT-29 (ATCC HTB- 38, Wesel, Germany) were dissolved in a total volume of 0.5 mL as a 1:1 mixture of phosphate buffered saline (PBS pH 7.4; GIBCO Life Technologies, Darmstadt, Germany) and Matrigel (BD Biosciences, San Jose, CA) and were injected subcutaneously into the left abdominal flank (2x10^6^ cells per rat) of athymic nude rats (n = 17; 7–8 weeks old, Harlan Laboratories Inc., Indianapolis, IN). Subcutaneous xenografts were allowed to grow to a size of 800 mm^3^ assessed by daily caliper measurements in three dimensions (a×b×c). Animals were randomly assigned to either the treatment (n = 10) or to the control group (n = 7) and imaged using a multimodality imaging protocol including ^18^F-FDG PET and subsequent perfusion MRI on day 0 and day 7. Animals were treated with the multityrosine kinase inhibitor regorafenib (10 mg/kg bodyweight, Bayer Healthcare AG, Leverkusen, Germany), or with volume-equivalent applications of the regorafenib solvent (Cremophor/Ethanol) day 1 through day 6 via gastric gavage using a dedicated 16-gauge curved buttoned cannula. Subsequent to imaging on day 7, animals were euthanized and tumors were explanted, fixed in formalin for immunohistochemical work-up.

### Multiparametric MRI protocol

Multiparametric MRI was performed at 3 Tesla on a clinical MRI scanner (Magnetom Verio, Siemens Healthcare, Erlangen, Germany) with the rats in supine position, using a four-channel small Flex Coil (Siemens Healthcare, Erlangen, Germany). For the morphologic and anatomic assessment of tumor growth T2-weighted MR images were acquired using a 2D Turbo Spin Echo sequence (TR/TE: 5470/91ms) with 0.3x0.3 mm in-plane resolution resulting in a matrix size of 192x192 and slice thickness of 1.5 mm. Additionally a fast view-sharing gradient-recalled echo time-resolved angiography with stochastic trajectories (TWIST) sequence [26] was started for acquisition of the pre-contrast baseline and subsequent contrast medium bolus tracking with high temporal resolution and acquisition of n = 300 datasets, resulting in an acquisition time of 10:13 min (Sequence details: TR/TE: 6.34/2.11 ms; flip angle 40°; matrix size 128x128; field of view 50 x 50 mm^2^; spatial resolution, 0.39 x 0.39 x 3.0 mm^3^). For standardized contrast media administration an automated bolus of 0.1mmol/kg body-weight of gadobutrol (Gadovist, Bayer Healthcare, Leverkusen, Germany) followed by a saline bolus of 0.5 mL was applied using a dedicated small animal contrast media injection system (Harvard Apparatus PHD 2000 Infuse/Withdraw, Holliston, MA).

### MRI data processing and kinetic analysis

**Fig 1:**
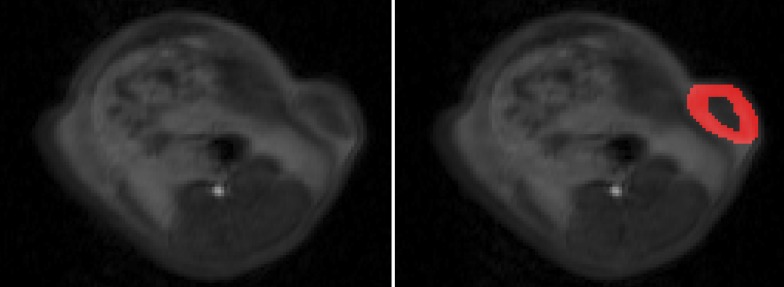
Representative axial TWIST MR images of a rat with a subcutaneous colon carcinoma xenograft over the left lateral flank after 7 days of regorafenib therapy. Note the hypointense tumor center corresponding to areas of beginning or present tumor necrosis and consecutively altered contrast media kinetics. For the assessment of tumor microcirculation a donut-shaped region-of-interest (red area) was drawn over vital tumor areas of the tumor periphery using semiquantitative AUC maps.

**Fig 2:**
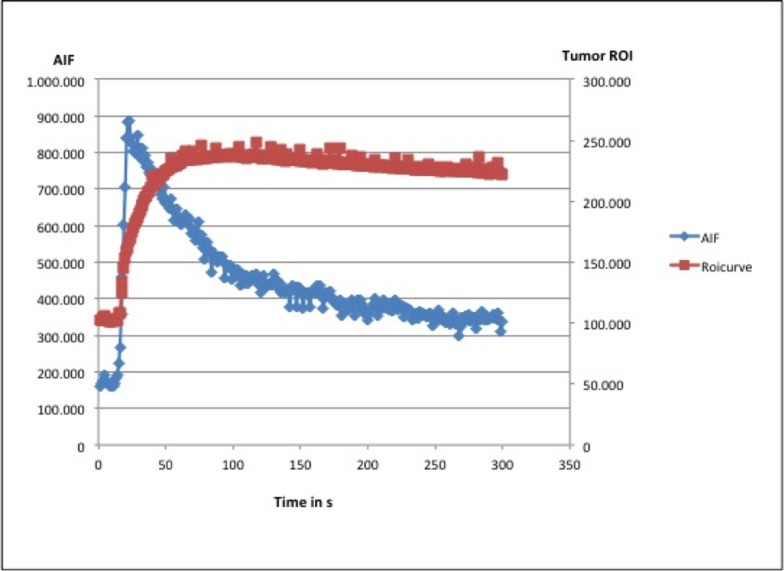
Representative signal-intensity vs. time curve for the assessment of contrast media kinetics in the tumor (red) and the inferior vena cava (blue).

Data sets were post-processed on an external workstation with PMI software (Platform for Research in Medical Imaging version 0.4) [27], written in house in IDL 8.3 (ITT Visual Information Solutions, Boulder, CO). A blood region of interest (ROI) was drawn into the lumen of the intrahepatic part of the inferior vena cava to assess an arterial input function (AIF) and a tissue region of interest was placed over the tumor periphery using semiquantitative AUC maps (Fig. 1). The tumor periphery was defined as the 3 mm outer rim of the tumor, as a representative region of viable tumor tissue less affected by elevated interstitial pressure and necrosis most present in the tumor center [28]. Signal intensity versus time curves (Fig. 2) were extracted for the AIF and the tumor tissue ROI and tracer concentrations were approximated in accordance to their relative enhancement (S/S_0_–1, where S is signal intensity and S_0_ is the signal intensity before arrival of the contrast agent). A two-compartment exchange model [29,30] was fitted to the MRI data and generated three independent model parameters: tumor plasma flow PF (mL/100mL/min), as a measure of tumor perfusion, tumor plasma volume PV (%), as a parameter of tumor vascularity, permeability-surface area product PS (mL/min/100 mL), as a parameter of tumor endothelial permeability and the tumor interstitial volume VE (%). Additionally ΔPF (PF-Baseline—PF-Follow-up), ΔPV (PV-Baseline—PV-Follow-up), PS (PS-baseline—PS-follow-up) and ΔTTB (TTB-baseline—TTB-follow-up) were calculated.

### ^18^F-FDG-PET

Small-animal PET was performed on a preclinical microPET scanner (Inveon, Siemens Healthcare AG, Erlangen, Germany). Each animal was placed in prone position inside a custom-built acrylic glass-imaging chamber for hygienic reasons and to allow for regulated anesthesia delivery. 60 min after manual intraperitoneal injection of ^18^F-FDG (50 MBq; ~500 μL), list-mode PET data were acquired for 30 min (15 min emission, 15 min transmission). Images were reconstructed using OSEM 3D algorithm with 128 x 128 matrix. All data were processed with an Inveon Acquisition Workplace (Siemens Healthcare, Erlangen, Germany). OSEM 3D image reconstruction algorithm was used as implemented in the software from the manufacturer. PET images were analyzed using Vinci 4.07 (http://www.nf.mpg.de/vinci3/). Maximum tumor uptake was measured in a corresponding tumor ROI over the tumor periphery in axial plane. Background was determined as mean uptake in a 7 mm diameter circular ROI in the right liver lobe in axial plane and tumor-to-background ratios (TTB) were calculated accordingly.

### Immunohistochemistry

The formaldehyde-fixed and paraffin-embedded tissue was stained with focus on various aspects of tumor pathophysiology, specifically microvascular density (CD-31), tumor cell proliferation (Ki-67) and apoptosis (TUNEL). Tissue samples were de-waxed and rehydrated following standard procedures including pre-heating at 60°C and washing in xylene substitute (Neo-Clear, Merck KgaA, Darmstadt, Germany) with rehydration in a graded series of ethanol (100%, 96%, 90% and 70% ethanol) followed by double distilled water. Subsequently, dedicated staining procedures were initiated.

### Ki-67 antigen staining

A *Ki-67*-specific monoclonal rabbit anti-human antibody (SP6, Abcam ab16667 1:100, Cambridge, United Kingdom) was used to quantify tumor cell proliferation. The tissue was de-masked in 1 x citrate buffer (pH = 6.0) (DAKO Diagnostika, Hamburg, Germany) using microwave irradiation at 600W. After washing the slides in ddH_2_O and TBS-Tween (0.05%) a multi-step kit (Dako EnVision+ System HRP (DAB), DAKO Diagnostika, Hamburg, Germany) was used for the staining, following the manufacturer’s instructions. Counterstaining was performed using Mayer´s Haemalaun (Merck KGaA, Darmstadt, Germany) and the slides were covered with Kaiser´s Glycerin Gelatine (Merck KgaA, Darmstadt, Germany). Results were quantified as the average number of proliferating cells in 10 random fields at 200x magnification.

### CD-31 antigen staining

For immunohistochemical assessment of tumor microvascular density, tumor sections were incubated with a polyclonal rabbit anti-CD31 primary antibody (Abcam ab28364 1:50, Cambridge, United Kingdom) overnight. Further work-up of tissue samples was performed using the EnVision+ System HRP (AEC) (DAKO Diagnostika, Hamburg, Germany) according to the manufacturer´s instruction. Counterstaining was performed using Mayer´s Haemalaun (Merck KgaA, Darmstadt, Germany) and slides were covered with Kaiser´s Glycerin Gelatine (Merck KgaA, Darmstadt, Germany). Tumor microvessels were quantified as previously described in the literature [31]. Results were quantified as the average number of endothelial cells in 10 random fields at 200x magnification.

### Statistical analysis

Continuous variables are presented as means with standard deviations. Comparison of MRI perfusion parameters, PET tumor-to-background-ratio and immunohistochemical values between the treatment and control group were performed using the Mann-Whitney-U-test. A Wilcoxon-signed-rank test was applied for intragroup comparisons of MRI perfusion and PET parameters between baseline (day 0) and follow-up (day 7). Correlations between MRI perfusion and PET TTB as well as between MRI perfusion and immunohistochemistry were evaluated by Spearman’s correlation coefficients. P-values <0.05 were considered statistically significant. All analyses were performed with SPSS software for Microsoft Windows (version 21.1, IBM, Armonk, NY).

## Results

The experimental protocol, including multimodality imaging and immunohistochemistry was successfully completed in n = 17 animals. The animals tolerated all procedures well, without adverse effects noted. At baseline no significant differences in tumor volume were present between the therapy and the control group. In the therapy group, mean tumor volume did not change significantly between day 0 (581 ± 131 mm3) and day 7 (780 ± 235 mm3; p>0.05). A significant increase of tumor volume was observed in the control group between day 0 (235 ± 30 mm3) and day 7 (442 ± 51 mm3; p<0.05).

### Perfusion MRI

**Fig 3:**
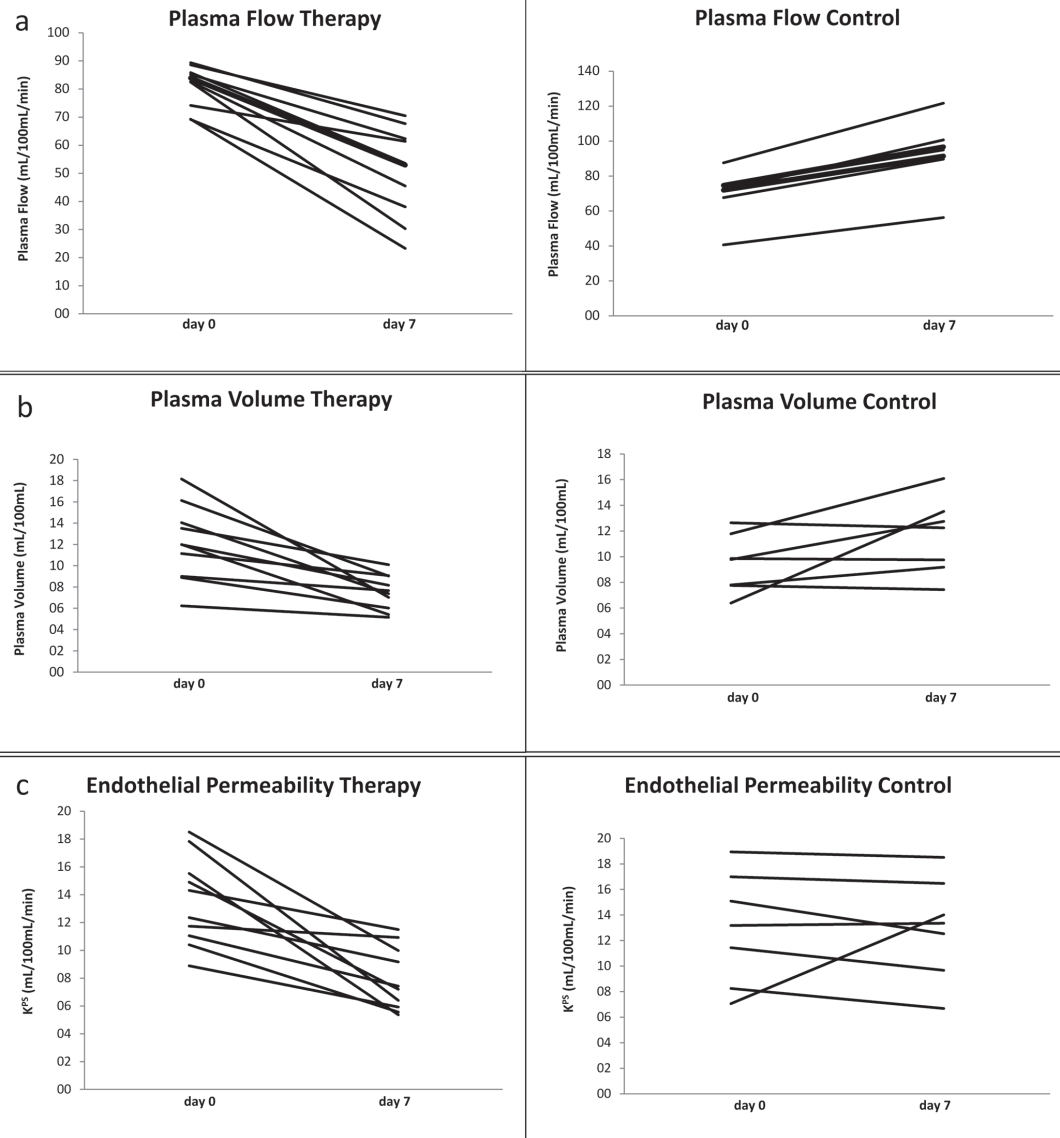
Line diagrams of the development of individual values of perfusion MRI parameters of tumor microcirculation in the therapy and the control group. **a.** Note the significant (p<0.01) unidirectional decline of tumor plasma flow (mL/100mL/min) in all tumors under regorafenib therapy and a significant unidirectional increase of tumor perfusion (p<0.03) in untreated control group between baseline (day0) and follow-up (day 7). **b.** Note the significant (p<0.01) unidirectional decrease of tumor plasma volume (%) in all tumors of the regorafenib-treated therapy group between day 0 and day 7 as well as non-uniform, non-significant (p>0.05) changes in control group. **c.** Note the significant (p<0.01) unidirectional decline of tumor endothelial permeability in all tumors under regorafenib treatment between baseline and follow-up as well as a non-uniform development of individual values with non-significant (p>0.05) changes of mean PS in the control group.

The two-compartment model fit the data well in all experiments with a representative arterial input function and a tumor fit. In the regorafenib-treated therapy group plasma flow significantly (p<0.01) declined from 81.1 ± 7.5 mL/100 mL/min at baseline to 50.6 ± 16.0 mL/100 mL/min at follow-up on day 7. In the control group a significant (p<0.03) increase of plasma flow was observed from 69.9 ± 14.3 mL/100 mL/min on day 0 to 93.1 ± 19.4 mL/100 mL/min on day 7. Individual values demonstrated an unidirectional decline of tumor perfusion in the therapy group and an unidirectional increase in the control group. Mean plasma volume decreased significantly (p<0.01) over the course of the experiment from 12.1 ± 3.6% at baseline to 7.5 ± 1.6% at follow-up. In the control group no significant change (p>0.05) of mean plasma volume was observed between baseline and follow-up (9.4 ± 2.3% to 11.6 ± 3.0%). Individual values for baseline and follow-up measurements demonstrate an unidirectional decrease of tumor vascularity in therapy group and non-uniform changes in the control group. In the therapy group a significant (p<0.01) decrease of permeability-surface area product (PS) from 13.6 ± 3.2 mL/100mL/min to 7.9 ± 2.3 mL/100mL/min between baseline and follow-up was observed. In the control group no significant (p>0.05) changes of the permeability-surface area product were observed (13.0 ± 4.4 mL/100 mL/min to 13.0 ± 4.0 mL/100 mL/min). Individual values also demonstrate an unidirectional decline of endothelial permeability in therapy group and non-uniform changes in the control group. Table 1 lists the individual perfusion MRI values for baseline and follow-up measurements and Fig. 3 the corresponding line diagrams.

**Table 1:** Individual perfusion MRI parameters of tumor microcirculation with tumor plasma flow (mL/100mL/min), plasma volume (%) and endothelial permeability-surface area product PS (mL/100mL/min) baseline and follow-up in the therapy and the control group.

Animal No.	**Plasma Flow** [mL/100mL/min]			**Plasma Volume** [mL/100mL]			**Endothelial Permeability-Surface Area Product PS** [mL/100mL/min]		
	Day 0	Day 7	Delta PF	Baseline	Day 7	Delta PV	Baseline	Day7	Delta EF
THERAPY GROUP									
1	84.0	53.0	-31.0	18.2	7.0	-11.1	15.5	5.4	-10.2
2	85.3	62.4	-22.9	9.0	7.7	-1.3	14.9	7.2	-7.7
3	69.2	38.1	-31.1	16.1	9.0	-7.1	17.8	6.4	-11.4
4	69.3	23.3	-46.0	12.0	8.2	-3.8	8.9	5.9	-3.0
5	82.5	30.3	-52.2	8.9	6.0	-2.9	14.3	11.5	-2.8
6	82.7	45.6	-37.1	12.0	5.4	-6.6	10.4	5.6	-4.8
7	88.6	70.5	-18.1	6.2	5.2	-1.1	11.1	7.4	-3.6
8	89.4	67.7	-21.7	13.5	10.1	-3.4	11.7	10.9	-0.8
9	85.9	53.6	-32.3	14.1	7.4	-6.7	18.5	10.0	-8.5
10	74.2	61.4	-12.8	11.1	9.1	-2.1	12.4	9.2	-3.2
Mean	81.1*	50.6*	-30.5^#^	12.1*	7.5*	-4.6^#^	13.6*	7.9*	-5.6^#^
SD	7.5	16.0	12.3	3.6	1.6	3.2	3.2	2.3	3.6
CONTROL GROUP									
1	40.6	56.2	15.6	6.4	13.5	7.2	8.3	6.7	-1.6
2	72.0	91.4	19.4	9.9	9.8	-0.1	7.1	14.0	7.0
3	67.7	89.7	22.0	9.8	12.8	3.0	17.0	16.5	-0.5
4	74.6	96.7	22.1	7.8	7.4	-0.3	13.2	13.4	0.2
5	87.6	121.7	34.2	12.6	12.3	-0.4	19.0	18.5	-0.4
6	74.7	95.0	20.3	11.8	16.1	4.3	15.1	12.5	-2.6
7	71.9	100.7	28.8	7.8	9.2	1.4	11.5	9.7	-1.8
Mean	69.9**	93.1**	23.2^#^	9.4	11.6	2.1^#^	13.0	13.0	0.0^#^
SD	14.3	19.4	6.3	2.3	3.0	2.8	4.4	4.0	3.2

Note the significant decrease of tumor microcirculatory parameters between day 0 and day 7 (*) as well as between the therapy and the control group (#).

* significant difference (p<0.01) between baseline and follow-up

** significant difference (p<0.03) between baseline and follow-up

^#^ significant difference (p<0.01) between therapy and control group

### ^18^F-FDG-PET

**Fig 4:**
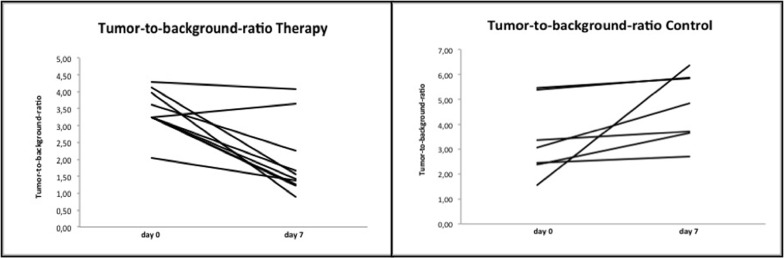
Development of individual values of tumor glucose metabolism, assessed by PET as tumor-to-background ratio (TTB), in the therapy and in the control group between baseline and follow-up. Note the almost unidirectional decline of individual TTB values with a slight increase of TTB in only one tumor of the regorafenib-treated therapy group as well as a significantly (p<0.01) decreased mean TTB between day 0 and day 7. In the control group a uniform increase of individual TTB values can be observed with a significant (p<0.03) rise of mean TTB in the untreated colon carcinoma xenografts.

In the regorafenib-treated therapy group ^18^F-FDG PET tumor-to-background-ratio (TTB) significantly (p<0.01) decreased from day 0 (3.4 ± 0.6) to day 7 (1.9 ± 1.1, Fig. 4). In the control group TTB increased significantly (p<0.03) from baseline (3.4 ± 1.5) to follow-up (4.7 ± 1.4) (Fig. 4). Individual values for baseline and follow-up TTB measurements are displayed in Table 2.

**Table 2:** Individual PET tumor-to-background (TTB) values in the investigated subcutaneous human colon carcinoma xenografts at baseline and follow-up in the therapy and in the control group.

Rat No.	Tumor-To-Background-Ratio (TTB)		
	Day 0	Day 7	Delta TTB
THERAPY GROUP			
1	4.0	0.9	-3.1
2	3.2	1.3	-2.0
3	4.1	1.6	-2.6
4	2.0	1.4	-0.7
5	3.3	1.2	-2.0
6	3.3	1.4	-1.8
7	3.3	1.7	-1.6
8	3.6	2.3	-1.4
9	3.2	3.7	0.4
10	4.3	4.1	-0.2
Mean	3.4*	1.9*	-1.5^#^
SD	0.6	1.1	1.1
CONTROL GROUP			
1	2.5	2.7	0.3
2	3.4	3.7	0.4
3	2.4	3.7	1.3
4	3.1	4.9	1.8
5	1.6	6.4	4.8
6	5.5	5.8	0.4
7	5.4	5.9	0.5
Mean	3.4**	4.7**	1.3^#^
SD	1.5	1.4	1.6

Note the significant (p<0.01) decrease of tumor TTB between day 0 and day 7 in the therapy group, as well as the significantly (p<0.01) increased glucose metabolism in the control group on day7.

*significant difference (p<0.01) between baseline and follow-up

** significant difference (p<0.03) between baseline and follow-up

# significant difference (p<0.01) between therapy and control group

### Immunohistochemistry

**Fig 5:**
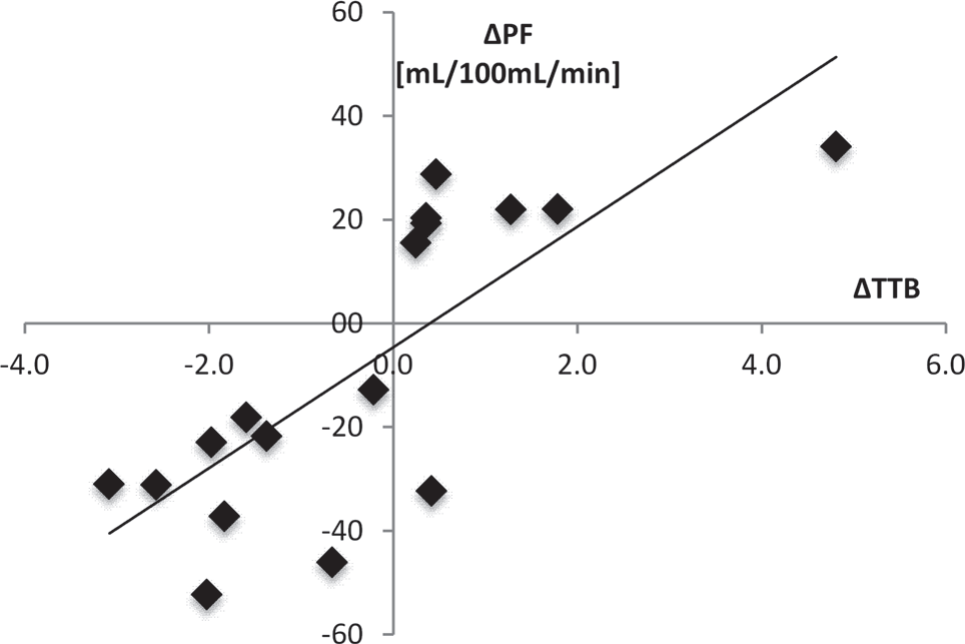
Representative tumor sections of human colon carcinoma xenografts with immunohistochemical stainings for tumor cell proliferation (*Ki-67*) and tumor microvascular density (CD-31) for the regorafenib-treated therapy (left column) and the control group (right column) *ex vivo*. Note the significantly (p<0.03) lower number of *Ki-67*-positive, proliferating cells (stained in brown) in the therapy group, consistent with anti-proliferative effects of regorafenib, as well as the significantly (p<0.03) higher number of CD-31-positive endothelial cells (stained in brown) in the untreated control group, consistent with anti-angiogenic effects of regorafenib.

Significant anti-angiogenic effects of regorafenib were observed in the investigated colon carcinoma xenografts with a significantly (p<0.05) lower tumor microvascular density, quantified by CD-31 stainings, in the regorafenib-treated therapy than in control group (7.0 ± 2.4 vs. 16.1 ± 5.9). Significant anti-proliferative effects of regorafenib were noted, with a significantly (p<0.03) lower number of proliferating cells in therapy than in control group (*Ki-67*, 434.0 ± 62.9 vs. 663.0 ± 98.3). Representative tumor sections with stainings for CD-31 and *Ki-67* are displayed in Fig. 5. Individual values of immunohistochemical stainings are displayed in Table 3.

**Table 3:** Individual values for the investigated immunohistochemical parameters of tumor microvascular density (CD-31) and tumor cell proliferation (Ki-67) in the therapy and control group.

Therapy Group		
Rat No.	CD-31	Ki-67
1	4.5	470.8
2	7.9	388.6
3	5.1	407.6
4	6.1	397.2
5	4.8	543.2
6	8.4	528.9
7	5.8	362.1
8	10.2	379.3
9	11.3	454.0
10	5.6	408.5
Mean	7.0*	434.0**
SD	2.4	62.9
Control Group		
1	14.7	842.8
2	22.5	679.6
3	5.7	581.5
4	17.8	566.2
5	14.3	583.6
6	14.1	670.3
7	23.3	717.3
Mean	16.1^*^	663.0^**^
SD	5.9	98.3

Note the significantly reduced tumor vascularity and tumor cell proliferation in the regorafenib-treated therapy group.

* significant difference (p<0.03) between therapy and control group

** significant difference (p<0.03) between therapy and control group

### Correlation between MRI perfusion parameters and ^18^F-FDG-PET

**Fig 6:**
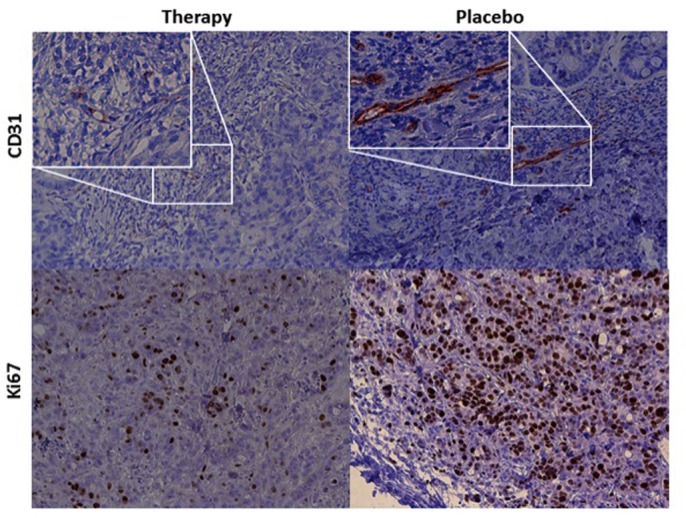
Representative line regression analysis for Spearman’s correlation coefficient between tumor plasma flow (ΔPF, mL/100mL/min) and tumor glucose metabolism (ΔTTB). Note the good and significant correlation between ΔPF and ΔTTB (r = 0.78, p<0.01) for the values of the therapy and the control group.

Good to moderate, significant correlations were observed between tumor plasma flow ΔPF and PET tumor-to-background ratio ΔTTB (r = 0.78; p<0.01) (Fig. 6), between ΔPV and ΔTTB (r = 0.67; p<0.01) and between ΔPS and ΔTTB (r = 0.75; p<0.01) (Table 4).

**Table 4:** Moderate to good and highly significant correlations between perfusion MRI parameters of tumor microcirculation including tumor plasma flow (ΔPF), tumor plasma volume (ΔPV) the tumor endothelial surface area product (ΔPS) with PET ΔTTB.

Correlation	Spearman r	p
ΔPF/ΔTTB	0.78*	< 0.01
ΔPV/ΔTTB	0.67*	< 0.01
ΔPS/TTB	0.75*	< 0.01

*significant correlations

### Correlation between MRI perfusion parameters and immunohistochemistry

ΔPF showed moderate, but significant correlations with CD-31, as a marker of tumor microvascular density (r = 0.51; p<0.05) and with *Ki-67*, as a marker of tumor cell proliferation (r = 0.57; p<0.03). Moderate but significant correlations were also observed between ΔPV and CD-31-stainings (r = 0.55; p<0.03) and between ΔPV and *Ki-67* (r = 0.67; p<0.01), as well as between ΔPS and CD-31 (r = 0.49; p<0.05) and between ΔPS and *Ki-67* (r = 0.57; p<0.03) (Table 5).

**Table 5:** Modest, but significant correlations between perfusion MRI parameters of tumor microcirculation and immunohistochemical markers of tumor microvascular density (CD-31) and tumor cell proliferation (*Ki-67*).

Correlation	Spearman r	p
ΔPF/CD-31	0.51*	< 0.05
ΔPF/Ki-67	0.57*	< 0.03
ΔPV/CD-31	0.55*	< 0.03
ΔPV/Ki-67	0.67*	< 0.01
ΔPS/CD-31	0.49*	< 0.05
ΔPS /Ki-67	0.57*	< 0.03

*significant correlations

### Correlation between ^18^F-FDG-PET parameters and immunohistochemistry

Modest, but significant correlations were observed between ΔTTB and CD-31 (r = 0.51; p<0.05), as well as between ΔTTB and *Ki-67* stainings (r = 0.56; p<0.03) (Table 6).

**Table 6:** Moderate, but significant correlations between PET tumor-to-background ratio (ΔTTB) and immunohistochemical markers of tumor microvascular density (CD-31) and tumor cell proliferation (*Ki-67*).

Correlation	Spearman r	p
ΔTTB/CD-31	0.51*	<0.05
ΔTTB/Ki-67	0.56	<0.03

*significant correlations

## Discussion

**Fig 7:**
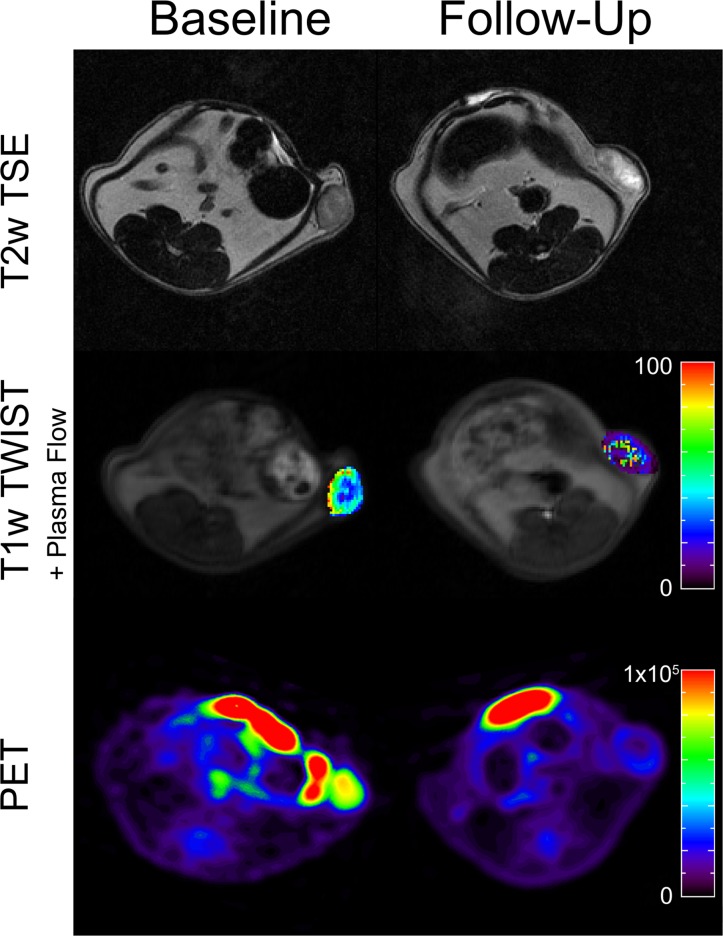
Representative axial images of the investigated human colon carcinoma xenografts including tumor morphology (T2w images, top row), tumor perfusion parameter maps (middle row) and ^18^F-FDG-PET (bottom row) before therapy (left column) and after a one-week treatment course of regorafenib (right column). Note the significant decrease of tumor plasma flow (mL/100mL/min) and tumor glucose metabolism (TTB) after regorafenib therapy between baseline and follow-up. The hyperintense central tumor regions on the T2-weighted image after therapy are consistent with increasing, therapy-induced tumor necrosis.

The present study evaluated the potential of functional imaging biomarkers of therapy response using a multimodality imaging protocol with perfusion MRI and ^18^F-FDG-PET for the non-invasive monitoring of regorafenib therapy effects on experimental colorectal carcinoma xenografts in rats. Results demonstrated that the multiparametric MRI/PET imaging protocol allowed for the early detection of regorafenib effects by measuring significant changes of tumor microcirculation and glucose metabolism under therapy (Fig. 7). In accordance with these results, immunohistochemistry revealed a significant reduction of tumor microvascular density and tumor cell proliferation under regorafenib therapy. Quantitative changes of tumor microcirculatory parameters showed significant correlations with changes of tumor glucose metabolism and with immunohistochemical parameters of tumor vascularity and tumor cell proliferation. These results support our hypothesis that perfusion MRI and ^18^F-FDG-PET could be clinically applicable for generating non-invasive imaging biomarkers for monitoring early changes in tumor pathophysiology under molecular cancer therapies.

### Perfusion MRI

During a one-week treatment protocol with regorafenib, a significant decrease of tumor perfusion, tumor vascularity and tumor permeability was observed, whereas a significant increase of tumor perfusion was detected in the control group. These results are in accordance with several pre-clinical and clinical studies in the literature [32–34], including Padhani et al who reported that tumor plasma flow quantified by dynamic, contrast-enhanced (DCE-) MRI decreased significantly following a one-week treatment protocol with the multityrosine kinase inhibitor sorafenib in an experimental model of breast carcinoma in mice [35]. The uni-directional decrease of all individual values of tumor microcirculation, including plasma flow, plasma volume and endothelial permeability surface area product, under regorafenib therapy is in support of our hypothesis that in the current experimental setting MRI parameters of tumor microcirculation can be reliably applied as non-invasive imaging biomarkers of therapy response to molecular cancer therapies *in vivo*. With regard to clinical translation, however, major unsolved issues still exist, as a reproducible assessment of quantitative parameters of tissue microcirculation using perfusion imaging is strongly dependent on standardized protocols of data acquisition and post-processing [3,36–39]. The lack of harmonization in perfusion imaging data acquisition and post-processing between imaging centers can be regarded as the prime obstacle for the broad implementation of functional perfusion MRI imaging into clinical practice over the last years. Although multiple initiatives have attempted, no broadly accepted consensus was reached so far.

### ^18^F-FDG-PET

In the same experimental sessions, concurrent ^18^F-FDG PET examinations were performed to acquire functional information on tumor glucose metabolism in the investigated colon carcinoma xenografts and to evaluate potential effects of the multityrosine kinase inhibitor regorafenib on ^18^F-FDG tumor-to-background ratio between liver and vital tumor tissue, a well-proven surrogate parameter of glucose metabolism. [40–42]. Quantitative assessments of tumor glucose metabolism using ^18^F-FDG-PET can be used as a surrogate endpoint to determine early treatment efficacy in established primarily cytotoxic tumor therapies (e.g. chemotherapy, radiotherapy) and novel molecular cancer therapies alike [41]. Kristian et al. reported in a study investigating triple-negative breast cancer xenografts in mice that ^18^F-FDG-PET can be applied for the assessment of therapy effects of the anti-VEGF antibody bevacizumab as early as 24h after first treatment [42]. In our study, a significant decrease of ^18^F-FDG tumor-to background ratio was observed in therapy group, while a significant increase of tumor glucose metabolism was detected in the untreated control group. Intraindividual comparisons of tumor ^18^F-FDG TTB and perfusion MRI parameters revealed good and significant correlations for the therapy and the control group. These findings are in accordance with Meier et al who reported in an experimental study of rhabdomyosarcoma xenografts under anti-angiogenic treatment that perfusion MRI parameters of tumor microcirculation showed correlations with glucose metabolism acquired by ^18^F-FDG PET [43]. The authors concluded that the correlating decrease of tumor microcirculation and glucose metabolism under treatment was most likely due to reduced tissue perfusion and tumor metabolism in early occurring pre-necrotic tumor areas.

A multimodality, multiparametric imaging protocol combining morphological and functional MRI with ^18^F-FDG-PET offers a comprehensive, multifaceted non-invasive characterization of the tumor microenvironment *in vivo* yielding information on microvascular and metabolic changes under treatment [17,44]. The acquisition of functional information on tumor microcirculation and glucose metabolism complements high-resolution morphological imaging of tumors with perspectives not only for early therapy monitoring, but also for pre-therapeutic treatment stratification of patients and evaluation of tumor heterogeneity in intensity modulated radiotherapy [45–47]. Additionally, the complementary information may provide a higher level of confidence for the stratification of therapy responders from non-responders in situations where the PET or MRI data alone is inconclusive. However, the combination of both modalities complicates the implementation of integrated imaging protocols and may be less feasible in clinical routine, also in the light of protocol length and examination time. Additionally challenges exist with regard to the implementation of standardized imaging protocols for MRI and PET, a problem that still remains unsolved for perfusion MRI data acquisition and analysis, but also present in PET where semi-quantitative SUVmax values are dependent on scanner type, detectors or acquisition protocol. The advent of integrated hybrid MRI/PET scanners to patient care will have to prove if routine examinations investigating functional information from both modalities significantly enhance diagnostic accuracy.

### Immunohistochemical validation

Immunohistochemical analysis revealed significant anti-angiogenic and anti-proliferative effects of regorafenib on colorectal carcinoma xenografts. In the treatment group the decrease of plasma flow,plasma volume and endothelial permeability correlated moderately, but significantly with a decline in microvascular density and tumor cell proliferation quantified by immunohistochemistry. The observed correlations between tumor microcirculation and immunohistochemical parameters of tumor pathophysiology may be explained by reduced tissue perfusion in pre-necrotic tumor areas [48] and a regress of the fraction of tumor vessels in the early stages of the treatment period [25]. Contrarily, Atkin and co-workers reported a paradoxical negative correlation between tumor microcirculatory parameters quantified by perfusion MRI and immunohistochemical assessments of tumor microvascular density (CD-31) in patients with rectal adenocarcinoma [49]. The reason for these variable results may be a lack of standardization with a large variety in MRI data acquisition and analysis protocols as well as immunohistochemical techniques. Although the observed correlations between perfusion MRI and immunohistochemistry in our study were only moderate, the results of our study are in support of our hypothesis that the investigated parameters of tumor microcirculation directly reflect processes of tumor pathophysiology and are therefore applicable as non-invasive imaging biomarkers of therapy response.

Our results are limited in several aspects. First, our multimodality imaging protocol was implemented on separate PET and MRI scanners. Although the time between both examinations was kept to a minimum in our study, an integrated imaging protocol on a hybrid MRI/PET scanner with synchronous acquisition of both parameters may offer higher data stability and broadened concordance between MRI and PET parameters due to leveled experimental conditions. Our study investigated only one tumor-therapy-combination in a heterotopic xenograft model of colon cancer with only limited translational relevance to orthotopic tumor pathophysiology in humans. For validation purposes of imaging results, only a small scope of immunohistochemical stainings were investigated with significant, but only moderate correlations to the acquired MRI parameters of tumor microcirculation. Nevertheless the selected immunohistochemical parameters can be considered representative for central aspects of tumor pathophysiology under molecular cancer therapy.

In conclusion, our results indicate that a multimodal, multiparametric perfusion MRI / PET imaging protocol allows for the early and reliable assessment of regorafenib therapy effects in the investigated experimental model of colorectal adenocarcinoma. Study results add further support to the hypothesis of a present biological relationship between functional parameters of tumor microcirculation and tumor cell glucose metabolism by the observed significant correlations between perfusion MRI and ^18^F-FDG-PET data. Beyond the individualized applicability of the parameters as imaging biomarkers of therapy response to molecular cancer treatments, the investigated multimodal, multiparametric perfusion MRI / PET imaging protocol offers a comprehensive, multi-faceted and non-invasive characterization of the tumor microenvironment *in vivo*. However, clinical investigations on integrated hybrid MRI/PET scanners will have to prove if routine examinations investigating functional information from both modalities significantly enhance diagnostic accuracy in patient care.
